# Laser-Induced Maculopathy and Outcomes After Treatment With Corticosteroids and Lutein

**DOI:** 10.7759/cureus.18470

**Published:** 2021-10-04

**Authors:** Anca I Marinescu, Caterina M Hall

**Affiliations:** 1 Ophthalmology, Moorfields Eye Hospital, London, GBR; 2 Ophthalmology, University College London, London, GBR

**Keywords:** visual acuity, retinal diseases, lasers, fovea centralis, adrenal cortex hormones

## Abstract

This case report presents treatment outcomes for a patient with accidental laser-induced retinal injury. A 30-year-old man was evaluated for a sudden decrease of vision and metamorphopsia in his left eye after staring at a laser in a nightclub five days before presentation. Eye examination showed left visual acuity of 6/18-2 unaided, which improved to 6/12-2 on the pinhole test. Dilated fundoscopy showed a yellow-orange foveolar lesion in the left eye. Optic coherence tomography (OCT) showed an alteration of foveal anatomy predominantly involving the outer retinal layers, hyper-reflective vertical bands, and large cystoid change at the inner retina. Foveolar thickness was increased to 397 µ. Treatment was initiated with oral corticosteroids (prednisolone 0.5 mg/kg/day). At the one-week follow-up, left visual acuity improved to 6/12+2. Hardly any cystic changes were noted, with fewer hyper-reflective bands and less disruption at the outer layer. Treatment with prednisolone was continued and lutein capsules (20 mg/day) were added. At three weeks, the patient reported a return to normal vision, with left visual acuity of 6/6-2 unaided. On OCT, near-complete restoration of the macular structure was visualized. Although these results show positive clinical outcomes with combined oral corticosteroids and lutein over a short time for a typical case of laser-induced maculopathy, further review is recommended to determine the ideal treatment regimen.

## Introduction

Lasers are an important tool in several sectors and are widely available for commercial purchase in the form of laser pointers and toys, among other products [1]. The potential for lasers to cause retinal injury is well documented [1]. Through a mixture of photochemical, thermal, and ionizing mechanisms, lasers can cause retinal damage that ranges from subclinical injury to involvement of all the layers in the foveal and parafoveal areas [1-3]. The extent of damage depends on several factors, including the duration of exposure and the properties of the laser, such as beam diameter, wavelength, and power output [2,3]. Based on these characteristics, four classifications describe the level of potential hazard [2-4]. Only classes 1 and 2 are commercially available in the United Kingdom; however, lasers with a higher classification can be purchased online [3-5]. Given that the demographic of accidental laser retinal injury is 11-34 years of age, the public health issues that arise with the use of this equipment are significant [6].

This report describes a case of an accidental laser injury in a 30-year-old man, the effects of the laser exposure, and the positive outcome after treatment with corticosteroids and lutein.

## Case presentation

A 30-year-old man was evaluated for a sudden decrease of vision in the left eye with metamorphopsia and an inability to focus. He recalled staring at a laser beam at a nightclub five days before the presentation but was unsure if the laser was the nightclub’s own equipment or if an individual at the club was using a laser pointer. He did not remember the duration of exposure to the laser. He had no previous history of amblyopia or squint and was otherwise in good health.

**Figure 1:**
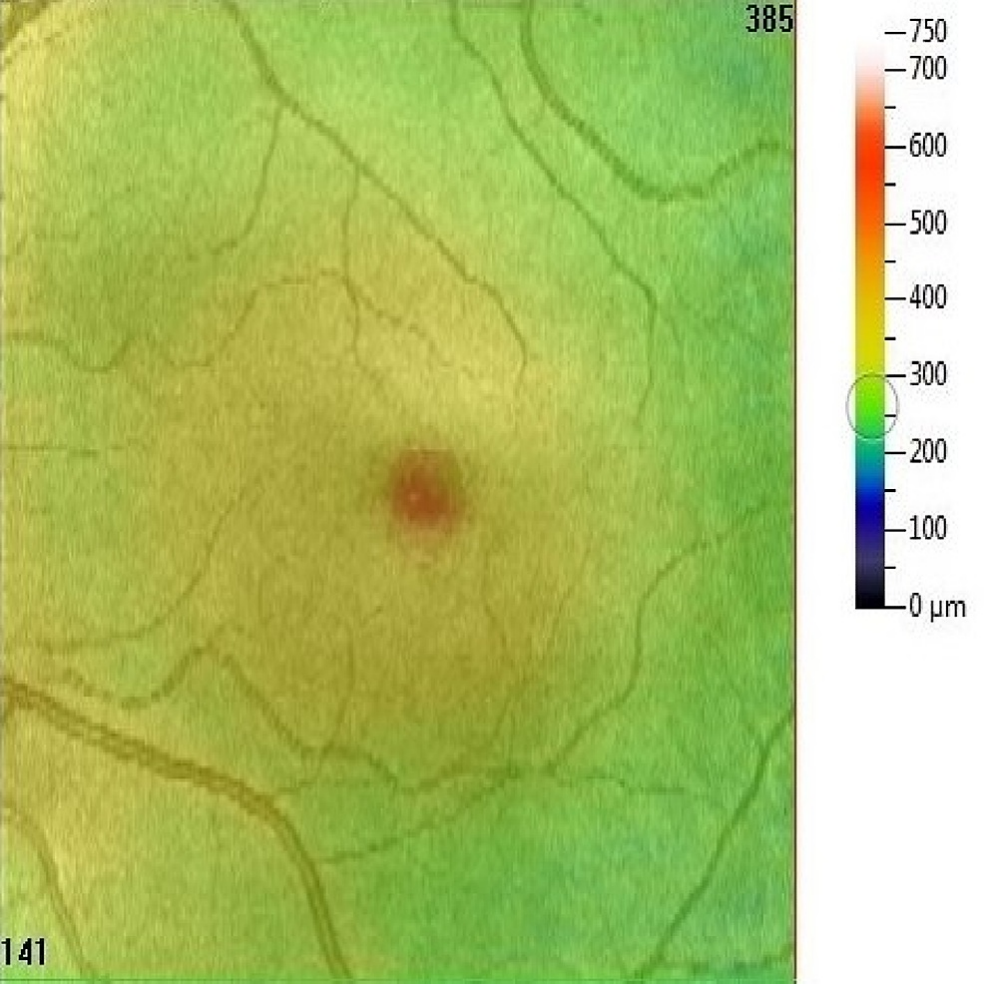
Left eye yellow-orange foveolar lesion at presentation.

At presentation, right visual acuity was 6/6 unaided and left visual acuity was 6/18-2 unaided, which improved to 6/12-2 on the pinhole test with difficulty. On 10-2 visual field testing, results for the right eye were normal, but the left eye showed a slight paracentral defect. No rapid afferent pupillary defect was noted. Ophthalmic examination showed unremarkable anterior segment, lens, and vitreous. Dilated fundoscopy showed a yellow-orange foveolar lesion in the left eye (Figure 1). The right eye was normal.

**Figure 2:**
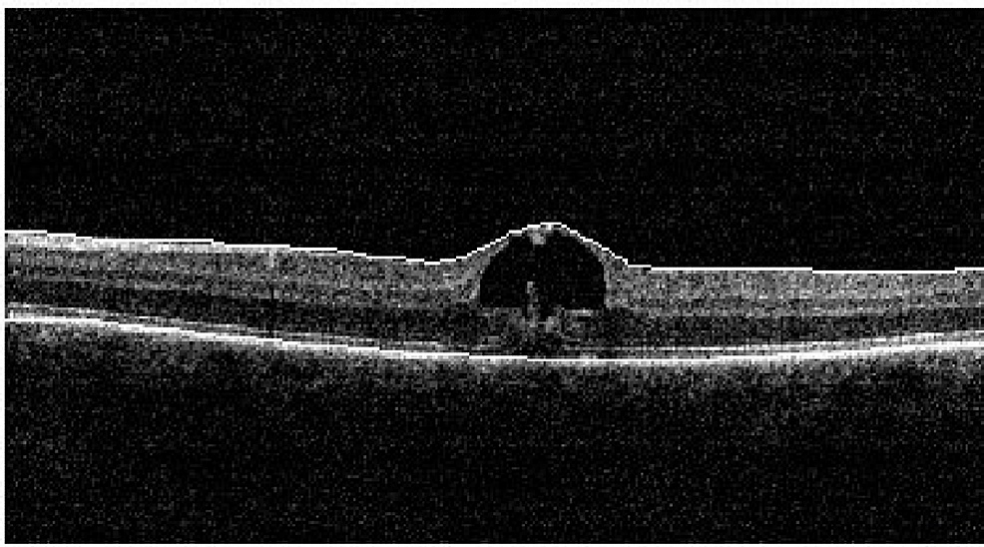
At presentation, OCT left eye disruption at the junction of the inner and outer segment of the photoreceptors and the inner aspect of the RPE, thin vertical hyper-reflective bands, and large cystoid change. Central foveolar thickness of 397 µ. OCT: optic coherence tomography; RPE: retinal pigment epithelium

**Figure 3:**
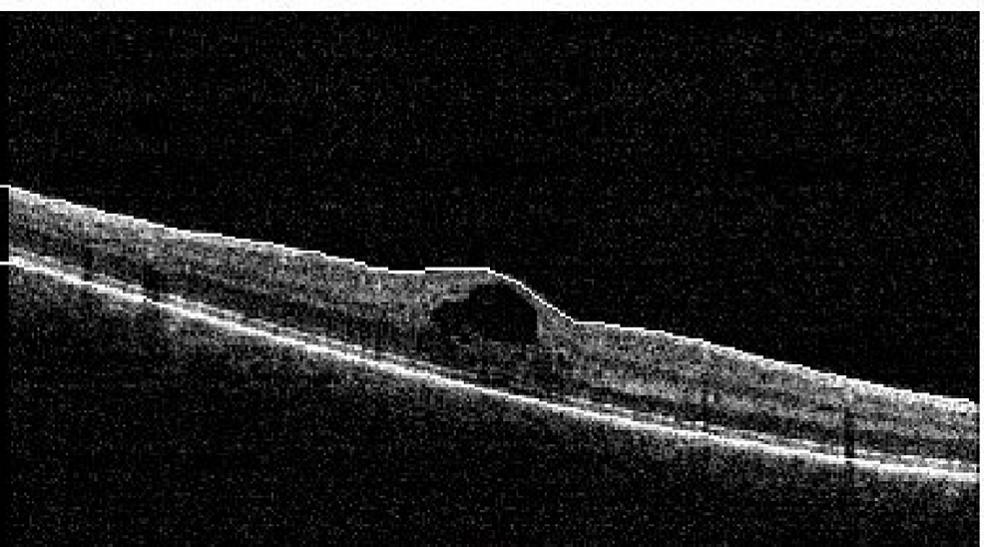
OCT of the left eye at presentation shows disruption at the junction of the inner and outer segment of the photoreceptors and the inner aspect of RPE, thin vertical hyper-reflective bands, and a large cystoid change. Central foveolar thickness is 397 µ. No features of central serous choroidal retinopathy are evident. OCT: optic coherence tomography; RPE: retinal pigment epithelium

Optic coherence tomography (OCT) showed an explosion-like alteration of foveolar anatomy involving the outer retinal layers and hyper-reflective vertical bands extending from the outer photoreceptor to the Henle layer (Figures 2, 3). Interestingly, a large cystoid change at the inner retina was also detected.

An inflammatory response was suspected and the patient was started on treatment with oral corticosteroids (prednisolone 0.5 mg/kg/day) as a fast-tapering regime. Because of the potential adverse effects of corticosteroids, the starting dose was lower than suggested in another study [7].

**Figure 4:**
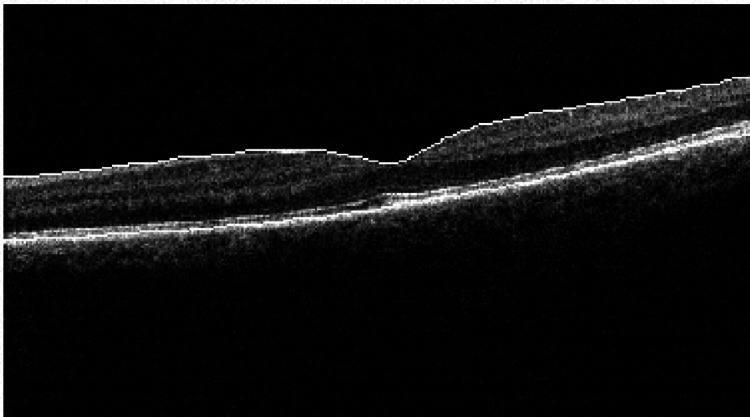
OCT of the left eye at the one-week follow-up shows a small defect in the outer layer, hyper-reflective bands, and a small cyst in the inner retina. Central foveolar thickness is 274 µ. OCT: optic coherence tomography

**Figure 5:**
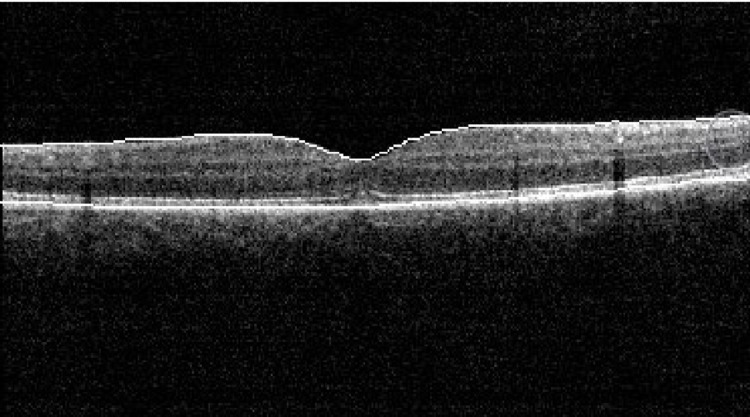
OCT of the left eye at the one-week follow-up shows a small defect in the outer layer, hyper-reflective bands, and a small cyst in the inner retina. OCT: optic coherence tomography

At the one-week follow-up, left visual acuity improved to 6/12+2 unaided. The disruption of the outer layer persisted, but hardly any cystic changes were evident in the left eye. Foveolar thickness was 274 µ (Figures 4, 5). The tapering regime with prednisolone was continued, and treatment with lutein capsules (20 mg/day) was added.

**Figure 6:**
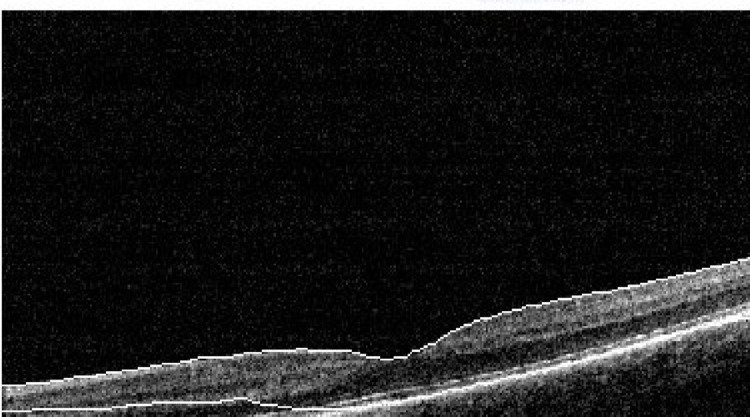
OCT of the left eye at the three-week follow-up shows restoration of the foveolar structure, no outer layer defect, a resolved cyst of the inner retina, and no vertical bands. Central foveolar thickness is 276 µ. OCT: optic coherence tomography

**Figure 7:**
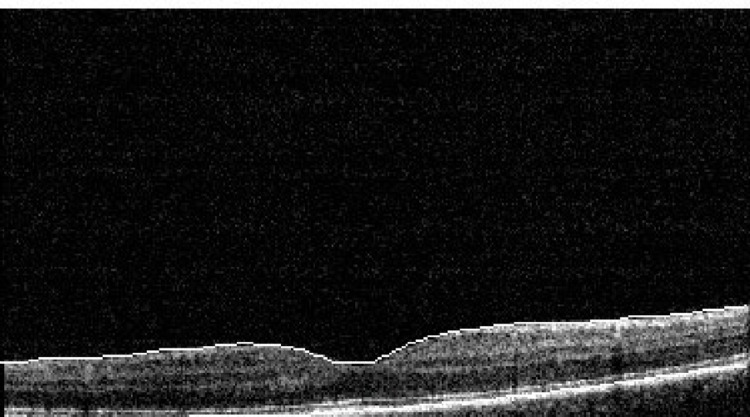
OCT of the left eye at the three-week follow-up shows restoration of the foveolar structure, no outer layer defect, a resolved cyst of the inner retina, and no vertical bands. OCT: optic coherence tomography

At the three-week follow-up, the patient reported that his vision was returning to normal. Left visual acuity improved to 6/6-2 unaided. On OCT, minimal disruption was noted, with the near-complete restoration of the macular structure. Foveolar thickness was 276 µ (Figures 6, 7).

Another evaluation was recommended after two months, but the patient did not present for follow-up.

## Discussion

In this case, the clinical history and OCT findings were typical of retinal injury caused by laser pointers reported in previous studies [6]. Laser pointers cause various injuries such as foveal granularity, perifoveal drusen-like deposits, pigment clamps, ring-shaped hyperpigmented lesions in the fovea, vitreous hemorrhage, or hemorrhages at different retinal layers [6,8]. Significant complications may be noted during follow-ups, such as a macular hole, choroidal neovascularization, and scars in the pigment epithelium [6,8]. The OCT findings of macular-induced injury may include vertical hyper-reflective bands, ellipsoid and external limiting membrane disruption, and/or hyporeflective cavities [6,8]. In the diagnosis of laser-induced maculopathy, OCT is an important tool because the effects of this injury may not be visible on slit-lamp examination [9]. One study classified retinal injuries as mild, moderate, and severe [10], with the following definitions: mild, “discrete outer retinal and retinal pigment epithelium” changes; moderate, “more diffuse changes” in these areas; and severe, “subfoveal loss” of anatomic structure and “hyperreflective bands in inner retinal layers” [10]. Using this classification, our case would be considered moderate based on the OCT findings.

Although no treatment regime for laser-induced maculopathy has been widely accepted [9], many studies show improved visual acuity and OCT findings after treatment with corticosteroids, attributed to a decreased release of cytokines and inhibition of retinal pigment epithelial proliferation [6,7,9,11,12]. In this case, the patient was started on prednisolone 0.5 mg/kg/day with a fast-tapering regime. One study that began the regime at 1 mg/kg/day found improvement compared with patients who did not receive treatment [7]. Another treatment reported as beneficial in some studies is lutein 20 mg/day for at least one month, administered for its antioxidant and anti-inflammatory effects along with its known protection in other macular diseases [9,13,14]. In this case, lutein was started at the one-week follow-up in addition to corticosteroids. However, it is difficult to determine whether the improvement can be attributed to the treatment because gradual improvement in untreated patients has been reported as well [4,6,9]. However, the improvement appears to occur over months to years, and, in some more serious cases, a degree of visual loss persists [10,15]. This case is interesting given the rapid improvement of visual acuity and the OCT findings over three weeks. Although this report is limited by the short follow-up period, it is noteworthy that the patient reported his experience of feeling his vision was back to normal and self-determined that further follow-up at two months was unnecessary.

Further review of the efficacy of various treatment regimens compared with observation alone would be beneficial in guiding future clinical practice. It would also be interesting to assess whether combined treatment with corticosteroids and lutein yields better outcomes than monotherapy.

This case presents several uncertainties regarding the laser injury event because the properties of the laser and the duration of exposure were unknown. Class 2 lasers are, in theory, the highest category available commercially in the United Kingdom [3,5]. These lasers should only cause injury after direct gaze for 10 seconds or more via the photochemical mechanism [1,3]. In dark surroundings, the consequent mydriatic pupil would increase the risk of injury; however, the blink reflex of up to 0.25 seconds and “aversion response” to the laser brightness should be protective [1,2,6]. Although we do not believe that the information in this case is sufficient to draw any conclusions, other studies have highlighted the lack of regulation and poor labeling standards online for laser devices [1,3,4,6]. We believe that a greater awareness of the dangers of lasers and increased regulation for online access are essential to prevent accidental injury in children and young adults in the future.

## Conclusions

This case study highlights the potential benefits of combined corticosteroids and lutein for the treatment of laser-induced maculopathy. These findings may be helpful to guide clinical treatment decisions in an acute care setting. Further comparative studies are needed to assess the efficacy of this treatment regime against other options.
